# Supplementary material for: Multi-Omics Characterization of Colon Mucosa and Submucosa/Wall from Crohn’s Disease Patients
Source: Int J Mol Sci. 2024 May 8;25(10):5108. doi: 10.3390/ijms25105108 (PMC11121447; doi:10.3390/ijms25105108)
Supplement: Supplementary file 1 [file ijms-25-05108-s001.zip › Supplementary Table S1.pdf]

Supplementary Table S1. Scoring for human clinical samples based on histology for severity of disease in mucosa and submucosa/wall (score 0-4, 4 most severe). Path comments specified presence of absence of tertiary lymphoid organs (TLOs) in histologic sections.

#	Group	Path score mucosa	Path score wall	Path comments
1	Inflamed	4 ulcerated	3	no TLOs
2	Inflamed	3	3	no TLOs
3	Inflamed	3-4	4	TLOs
4	Inflamed	3	3	no TLOs
5	Inflamed	4	4	TLOs
6	Inflamed	3	4	TLOs
7	Inflamed	4	4	TLOs
8	Inflamed	4	4	no TLOs
9	Inflamed	4	4	TLOs
10	Inflamed	3	4	TLOs
1	Non-inflamed	0	0	
2	Non-inflamed	0	0	
3	Non-inflamed	0	0	
4	Non-inflamed	0-1	0-1	
5	Non-inflamed	1	1 GALT	
6	Non-inflamed	2	0	
7	Non-inflamed	2	1 GALT	
8	Non-inflamed	1		
9	Non-inflamed	1	1	
1	Normal	0	0	
2	Normal	0	0	
3	Normal	1	0	
4	Normal	0	0	
5	Normal	0-1	0	
6	Normal	0	0	
7	Normal	1	1	
8	Normal	0	0	
9	Normal	1	0	
10	Normal	1	0	
