# Supplementary figures and images for: Multi-Omics Characterization of Colon Mucosa and Submucosa/Wall from Crohn’s Disease Patients
Source: Int J Mol Sci. 2024 May 8;25(10):5108. doi: 10.3390/ijms25105108 (PMC11121447; doi:10.3390/ijms25105108)

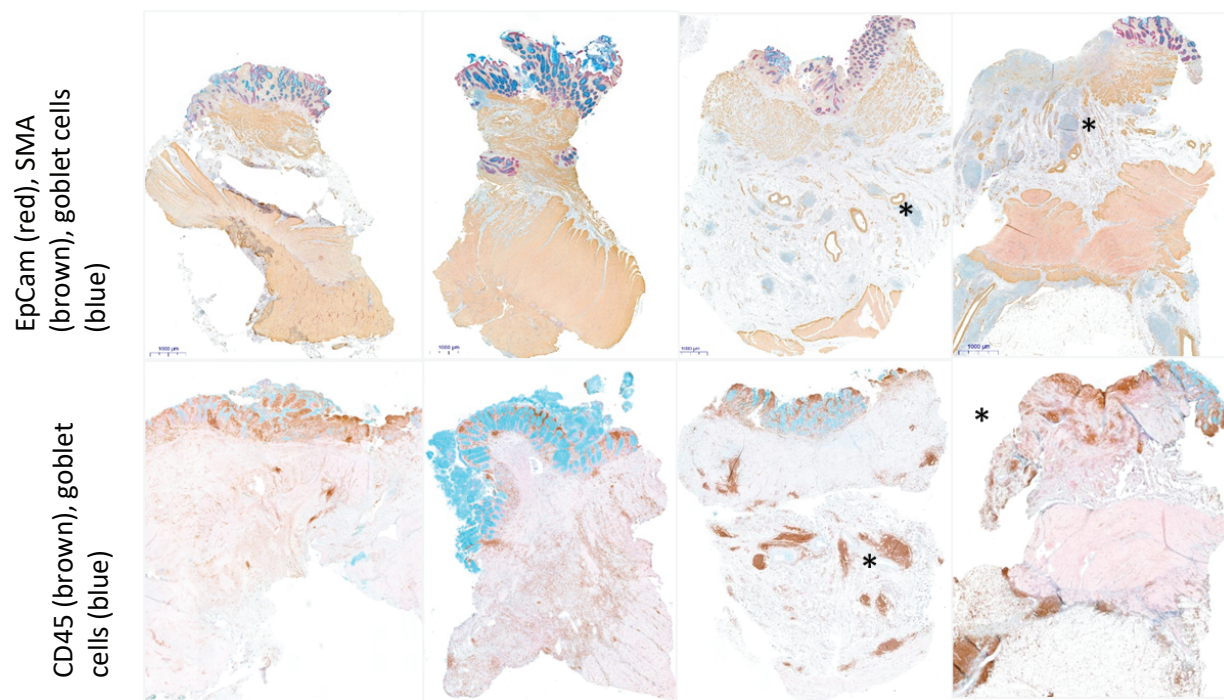

**Figure S1:** Pathological alterations in Crohn's disease mucosa and wall.

Supplement: Supplementary file 1 [file ijms-25-05108-s001.zip › Supplementary Figure S1.pdf]
